# Fear of cancer recurrence trajectory during radiation treatment and follow-up into survivorship of patients with breast cancer

**DOI:** 10.1186/s12885-018-4908-2

**Published:** 2018-10-20

**Authors:** Y. Yang, J. Cameron, C. Bedi, G. Humphris

**Affiliations:** 1 Department of Psychiatry and Psychology, Southern Medical University Nanfang Hospital, Guangzhou, Guangdong China; 2 Division of Population and Behavioural Sciences, School of Medicine, University of St Andrews, North Haugh, St Andrews, Fife UK; 3 Edinburgh Cancer Centre, Western General Hospital, Crewe Road, Edinburgh, UK

**Keywords:** Communication, Fear of recurrence, Longitudinal, Radiation treatment, Trajectory

## Abstract

**Background:**

Fear of cancer recurrence (FCR) has been shown to be higher in patients treated with external beam radiotherapy (RT) compared to those untreated. However, little is known about the dynamics of patient’s FCR during and after RT. The aim of this study was to examine FCR levels in a longitudinal panel design with breast cancer patients receiving RT.

**Methods:**

Consecutive newly-diagnosed breast cancer patients (*n* = 94) attending a single cancer centre were invited to complete a 7-item FCR scale (FCR7) that was collected weekly by paper instrument and at a follow-up phone call 6–8 weeks after completion of RT. Descriptive statistics, and Latent Growth Curve Modelling (LGCM) were utilised to analyse the data.

**Results:**

Women who were younger, single/separated, had chemotherapy, had extra boost radiation treatment, taking Herceptin and treated by 4-field technique reported higher recurrence fear at baseline. There was strong evidence of substantial variation in the trajectory of FCR (*z* = − 3.54, *p* < .0001). The average trajectory of FCR over RT was negative (unstandardized estimate = − 0.59) and associated with FCR follow-up level (standardised estimate = 0.36, *z* = 3.05, *p* < .002), independent of baseline recurrence fears.

**Conclusion:**

Patients vary in their trajectory of recurrence fears over RT which predicts FCR approximately 2 months following treatment. Review appointments by therapy radiographers presents an opportunity to intervene in FCR trajectories.

**Trial registration:**

ClinicalTrials.gov: NCT02599506. Prospectively registered on 11th March 2015.

## Background

Breast cancer (BC) is the second most common cancer in the world and the most frequent diagnosed cancer among women [1]. The majority of patients diagnosed with the disease undergo surgery, and a proportion receive chemotherapy and radiotherapy (RT). These treatments are frequently associated with significant psychosocial difficulty across the lifespan [2]. Fear of cancer recurrence (FCR) is one of the most common and aversive psychological phenomena among breast cancer patients [3]. It can be a concern for patients immediately after diagnosis or treatment and has been shown to remain stable for years [4].

Studies have shown that cancer patients “22 % to 87 % reported moderate to high degree of FCR (on average 49 %)” [5], and 55–90% of breast cancer patients report FCR throughout survivorship [6]. Cancer patients who suffer from high FCR report negative behaviour change (e.g. excessive personal checking behaviours and avoidance behaviour) [7], increased health service use [8], inability to plan for the future including work return [9] and significant psychological distress, such as depression, anxiety and post-traumatic stress symptoms [10–13].

A systematic review of 130 studies found that a number of factors were associated with FCR [5]. Evidence showed that those at greater risk of FCR were survivors diagnosed at a young age, female gender, and with higher education level. Women diagnosed with cancer before age 50 were more likely to suffer from FCR compared with their older counterparts [14]. Various clinical factors, such as having had a mastectomy or chemotherapy, and having more physical symptoms have also been identified as strong predictors of higher FCR. However, these findings are not always consistent [15–19].

Radiotherapy is a treatment frequently used for cancer patients involving the use of high-energy radiation [20]. Almost a half to two-thirds of cancer patients will have radiotherapy as part of their treatment plan (adjuvant treatment), and almost 75% of patients who received radiotherapy are treated to cure the cancer, rather than to relieve symptoms such as pain [21]. Radiotherapy is delivered in two ways – external to the body by a linear accelerator (external-beam radiation treatment, RT) or within the body by judicious siting of radioactive seeds/implants (brachytherapy, BT). According to the latest data, about 88% of patients received RT while the remaining 12% of patients received BT [20, 21].

To date, although studies have reported that cancer patients may suffer from different psychological problems such as anxiety, depression as well as psychological distress, in the course of RT [22, 23], there have been few studies investigating the relationship between patient’s FCR and the receipt of RT. A previous systematic review by Simard et al. [5] revealed a weak to moderate relationship between cancer treatment type (surgery/ chemotherapy/ radiation treatment) and FCR, and a recent meta-analysis [24] by our group, confirmed a weak association between RT and FCR, however, the correlation was found nonsignificant in breast cancer patients.

One study [25] has investigated FCR development over the first 6 months from surgery but does not focus specifically on the final stage of treatment (radiotherapy) where FCR first develops in earnest according to anecdotal reports. A longitudinal study by Manne et al. [26] found that almost half of the participants diagnosed with gynaecological cancer continued to experience a high level of FCR (high-stable) 6 months after diagnosis, and about 25% of the patients reported decreasing FCR over time (high-decreasing) while the remaining 25% of the sample reported consistently low FCR (low-stable) throughout the 6-month period post diagnosis. A recent report from the Netherlands followed patients soon after primary breast cancer surgery and tracked FCR at baseline, 6 months and 18 months follow up [27]. They found that FCR levels were stable for the first 6 months but at 1.5 years increased in younger compared to older patients. These studies add important information on the pattern of FCR following diagnosis but do not concentrate, we believe, on a critical phase during the patients’ time course namely at the point when RT is provided, and subsequent in the short to medium term, after the patient is discharged, from active hospital treatment. Patients during RT typically report symptoms of pain, skin problems and fatigue [28, 29]. According to Leventhal’s self-regulation model [30] some patients may misinterpret these symptoms to indicate disease progression rather than eradication, hence reflected in FCR increase over the course of treatment. No study has concentrated explicitly on FCR assessment at the RT treatment phase and soon thereafter. Neither has attention been focused on the different levels of fractionation received by patients with breast cancer. The majority of patients at the specialist cancer unit receive ‘routine’ RT treatment of 15 sessions. Patients with tumour margins that are less distinct are treated with the addition of RT with typically 4 ‘booster’ sessions, whereas DCIS patients with non-invasive disease receive 23 sessions. The clinical implications for patients’ FCR levels is unknown. To investigate therefore this stage in the patients’ ‘journey’ may assist our understanding of the development of FCR. Therefore, longitudinal studies on samples using a validated measure of FCR with cancer patients are needed during this phase of the treatment pathway [31, 32].

### Aim

To investigate the behaviour of FCR across various demographic and clinical related variables and the time course of RT.

### Objectives

1. To determine the effect of age, marital status, socio-economic status, chemotherapy and radiotherapy treatments, and self-reported comorbid conditions on initial FCR levels.
2. To test the predictive ability of initial FCR and its trajectory on 6–8 week follow up FCR level.

## Methods

### Participants and settings

All breast cancer patients were recruited from NHS Lothian, Edinburgh Cancer Centre (ECC), Western General Hospital. Patients were eligible if they were female; above 18 years of age; scheduled to undergo RT on the breast (+/− lymph nodes); and able to read, write and understand English. Patients were excluded if they were male; deaf or required translators, under age; or receiving palliative treatment. Patient participation was voluntary. All data were collected from March to September 2016.

### Instruments

A study specific set of questions (demographic/treatment received sheet) were formulated to assess age (in years), marital status, solitary living or with other(s), education level, occupational status, treatment type and technique, use of chemotherapy and/or Herceptin and self-rated co-morbidity.

#### Fear of recurrence scale (FCR7)

This measure was developed to assess recurrence fears and consists of items drawn from other FCR scales. It has good psychometric qualities and has been used with patients with breast, colorectal and head and neck cancer [33]. Scores range from 6 (minimum) to 40 (maximum), providing an effective range of 34 units [34]. The validation data set was collected prior to this study at the Edinburgh Cancer Centre including patients with breast cancer (*n* = 206) and provides reference levels of FCR for this scale. The reliability of the scale is good with an internal consistency of 0.92 (95%CI: 0.90, 0.94) and evidence for validity [34].

### Procedure

The University of St Andrews and the NHS East of Scotland Research Ethics Committee (NHS Lothian) examined and approved the study (NRES No.: 13/ES/0015). Study was registered on ClinicalTrials.gov (ID No.: NCT02599506). All new patients diagnosed with breast cancer attending the ECC during January to May 2016 were approached. During the patient’s pre-RT CT scan visit, a clinical staff (radiographer, JC) first introduced the study to the patient. For those women showing interest in participating, the staff member introduced the patient to the research assistant (YY), who met with the patients, explained the study in detail, and determined eligibility. Information sheet was provided to the patient during the initial research meeting. Written informed consent, demographic/treatment received information and baseline FCR7 total scores were obtained on the patient’s first treatment day (FCR7 Week 1). Throughout the period of RT, patient was asked to complete a weekly FCR7 scale (FCR7 Week 2 & FCR7 Week3). On the final day of RT, patient was asked to complete the FCR7 scale again (FCR7 Week4). Then, 6–8 weeks after the end of the treatment, all participants were contacted by telephone to give their FCR7 ratings once again (FCR7 Follow-up).

### Data analysis

Descriptive statistics were utilized to characterize all study covariates overall and FCR scores. Bivariate associations were investigated between sociodemographic, clinical/treatment factors and baseline recurrence fear. Individual and group average curves (routine, boost, and DCIS groups) were plotted over the period that patients were treated. Then, latent growth curve modelling (LGCM) for longitudinal data was developed to test the relationship of FCR over the time course of the treatment [35–37]. Of specific interest was the association of the initial FCR level (i.e. intercept) and the trajectory (i.e. slope) with the outcome FCR assessed at 6–8 week follow-up. A linear growth trajectory was proposed (i.e. 0–1–2–3). The limited sample size reduced our ability to test more complex models. MPlus uses full information maximum likelihood (FIML) estimation to exploit comprehensively all data points. Statistical analyses were conducted using SPSS Analytics software v.24 [38], and MPlus8.0 [39]. Alpha was 0.05 for all tests (2-sided).

## Results

### Recruitment and data collection

**Fig. 1:**
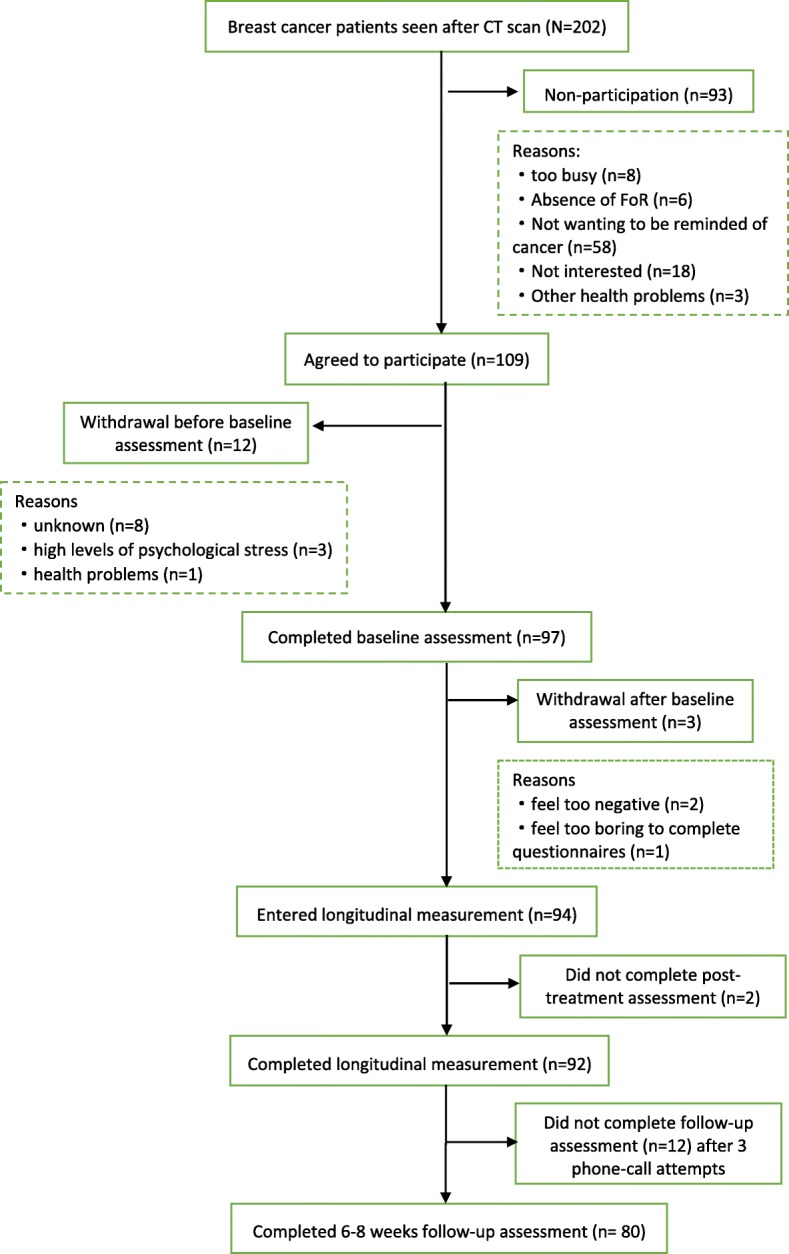
Patient recruitment flowchart

Patient recruitment flowchart was showed in Fig. 1. A total of 202 patients were approached after CT scan and 93 patients refused to participate in the study. The major reasons for refusal were: not wanting to be reminded of cancer (62%); not interested (19%); or too busy (9%). Finally, the total number of breast cancer patients enrolled in the study was 97 (response rate, 48%). There were 68 breast +/− lymph nodes (routine treatment), 24 breast +/−lymph nodes with boost treatment and the remaining 5 were DCIS patients who had breast treated alone. Three participants in the routine group withdrew from the study after baseline assessment (data destroyed), and two patients (one in routine group, and one in boost group) failed to complete post-treatment measurements. Of the 92 patients completing all assessments during the treatment phase, 12 were lost to follow up after 3 attempts to contact by telephone. A drop out analysis demonstrated that on all demographic, treatment and initial FCR levels the patients not followed up were not significantly different (*p* > 0.3) from patients with complete data. The exception was that 52% of patients with full data claimed to have an additional medical problem, whereas only 20% of the non-followed up patients reported initially they had this difficulty (*p* = 0.05).

### Patient characteristics

Table 1 displays the patients’ baseline sociodemographic, clinical/treatment characteristics. The mean age was 57.9 years (SD = 11.5, range: 28 to 85). On univariate analysis, fear of cancer recurrence was associated with patient’s current age (*p* < .001) and marital status (*p* < .005). Women who were younger and separated reported higher cancer recurrence fear. Baseline mean fear scores did not differ based on other sociodemographic factors, such as education level, living alone vs with others and occupational status. Other effects were demonstrated with radiation treatment type (*p* = .006), treatment technique (*p* = .003), adjuvant chemotherapy (*p* < .001) and Herceptin utilisation (*p* < .005). Patients who had extra boost radiation treatment and those treated by 4-field technique reported higher fear of recurrence. There were no significant differences in baseline mean fear scores between subjects with vs. without self-reported co-morbidity.

**Table 1:** Participant’s clinical and sociodemographic characteristics (*N* = 94)

Study Characteristic	Value	N (%)	Mean FCR (SD)	*P*
Sociodemographic				
Age	< 50	21 (22.3)	21.6 (8.7)	.0001***
	51–70	63 (67.0)	16.3 (6.3)	
	> 70	10 (10.6)	11.0 (6.2)	
Marital Status	Single	6 (6.4)	23.5 (11.3)	.005**
	Married	58 (61.7)	16.5 (6.4)	
	Separated	2 (2.1)	28.0 (4.2)	
	Partnered	12 (12.8)	18.1 (8.4)	
	Widowed	10 (10.6)	11.1 (5.3)	
	Divorced	6 (6.4)	17.8 (7.3)	
Patient Live With	Live Alone	17 (18.1)	16.9 (9.3)	.878
	Live with Partner	68 (72.3)	16.7 (6.7)	
	Live with Friend(s)	2 (2.1)	21.0 (12.7)	
	Live with Children	7 (7.4)	17.6 (9.8)	
Education Level	Left School at 16	50 (53.2)	16.4 (7.6)	.545
	Left School at 18	14 (14.9)	18.9 (6.4)	
	Have a University Degree	30 (31.9)	16.8 (7.8)	
Occupational Status	Full-time Employed	29 (30.9)	17.9 (7.2)	.203
	Part-time Employed	17 (18.1)	19.2 (9.7)	
	Retired	35 (37.2)	14.6 (6.5)	
	Temporarily unemployed	1 (1.1)	22.0	
	Housewife	12 (12.8)	17.8 (6.1)	
Clinical factors				
Rx Type	Routine	65 (69.1)	15.8 (7.3)	.006**
	Routine + Boost	24 (25.5)	20.9 (6.7)	
	DCIS	5 (5.3)	13.0 (6.0)	
Rx Technique	2 Field	74 (78.7)	16.1 (6.7)	.003**
	3 Field	9 (9.6)	15.0 (8.0)	
	4 Field	11 (11.7)	24.0 (8.4)	
Chemotherapy	Yes	39 (41.5)	20.4 (8.1)	.0001***
	No	55 (58.5)	14.5 (6.0)	
Taking Herceptin	Yes	8 (8.5)	23.9 (6.2)	.005**
	No	86 (91.5)	16.3 (7.3)	
Other Med Problems	Yes	44 (46.8)	15.4 (6.7)	.060
	No	50 (53.2)	18.3 (7.9)	

Recurrence Scores; Rx: Radiation treatment

**p ≤ .005, ***p ≤ .0005

The average FCR levels for the sample across the 4 weekly ratings as 17.2 (SD 7.4) at baseline to 14.5 (SD 7.0) at week 4. The intervening weeks 2 and 3 returned mean FCR levels of 15.6 (SD 7.6) and 15.0 (SD 7.4). The range of values of the mean total scores remained virtually at maximum across the weeks (i.e. 33 or 34). The published normative mean value of the FCR7 with patients with breast cancer (*N* = 206) was 16.96 (SD 6.9) and with a 90% cut-off of 27 [34]. Our sample of participants rated as 11, 9, 7, 7% over the cut-off respectively for the 4 weekly ratings [34].

### FCR trajectory

**Fig. 2:**
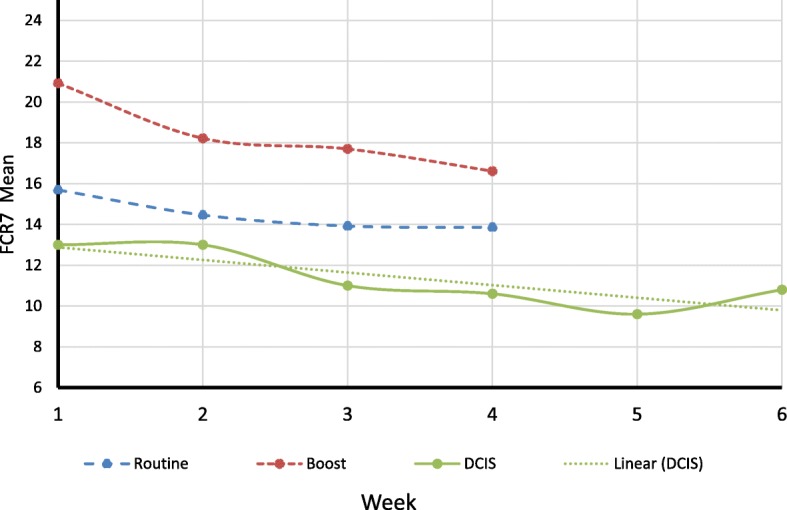
Weekly FCR7 trajectories for the 3 treatment groups: Routine (long dash), Booster (short dash), DCIS (solid line with dotted linear regression line fitted)

**Fig. 3:**
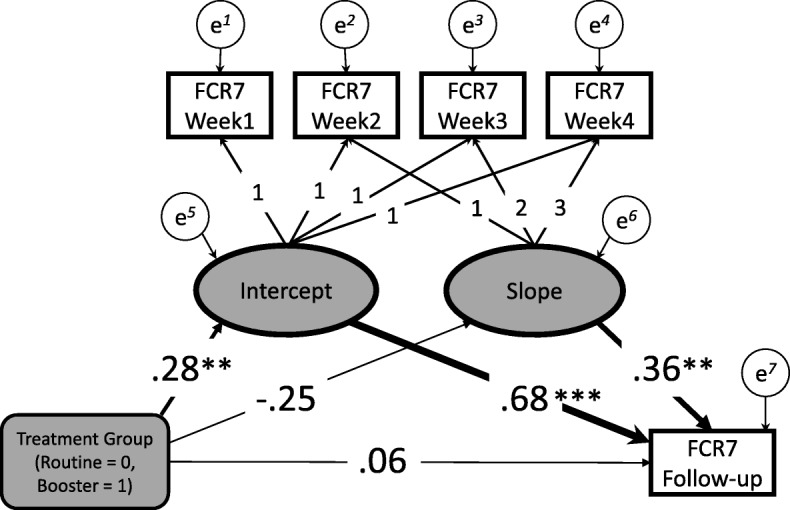
Latent Growth Curve Analysis model results. Latent variables shown as ellipses and raw scale values (FCR) in rectangular boxes. FCR7 Week 1 to FCR7 Week 4 denote FCR7 total scale values for the weekly ratings, FCR7 Follow-up denotes FCR7 total at 6–8 week follow up. Treatment variable in grey-shaded rounded box. Residuals indicated by circles. Arrows show model fitted. Standardised coefficients presented. Significance of structural model parameters shown using conventional symbols (** = *p* < 0.01; *** = *p* < .001) with width of path expanded according to significance for ease of interpretation

The group average FCR curves over RT were plotted for each of the three treatment groups (Fig. 2). Patients in routine group, boost group and DCIS group were treated 15 times, 19 times and 25 times, respectively. We concentrated our analysis of trajectories on the major two treatment groups (*n* = 87); routine (*n* = 64) and boost (*n* = 23). Prior to fitting the LGCM the covariances of the four weekly FCR totals (FCR7 Week 1 to FCR7 Week 4) were inspected. As illustrated by Wickrama et al. we compared the range of covariances in adjacent pairs of weekly observations (e.g. Week1 and Week2, etc.) with non-adjacent pairs (e.g. Week1 and Week3, etc.) [36]. Ratings assessed closer together should exhibit higher values according to Wickrama’s thesis. This was confirmed as shown by the adjacent range values of 47.7 to 51.4 compared with the non-adjacent range values of 41.2 to 47.9 and supporting significant slope variation for explanation within the data structure (page 19) [36]. On fitting the model it was confirmed that there was strong evidence of substantial variation in the trajectory of FCR (*z* = − 3.54, *p* < .0001). The average trajectory of FCR over RT was negative (unstandardized estimate = − 0.59) and associated with FCR follow-up level (standardised estimate = 0.36, *z* = 3.05, *p* < .002), independent of baseline recurrence fears (Fig. 3). The intercept (or baseline) was the strongest predictor of FCR follow-up level (standardised estimate = 0.68, *z* = 10.32, *p* < .0001). The overall fit of the model was good (CFI = 0.97, SRMR = 0.066).

## Discussion

This is the first study to examine in detail FCR levels over the time course of RT and use the behaviour of initial levels and its trajectory to predict follow up FCR. The results of the initial FCR ratings at the start of RT confirmed previous reports, namely that FCR was less with older compared with younger patients; that chemotherapy and Herceptin use was associated with increased FCR [5]. Most women experienced a decline in fear during and after RT. However, there was considerable variation of trajectories observed that warranted explanation. Studies reveal that RT-induced side effects, such as pain, tiredness and skin reaction, are common and contribute to the symptom burden [40]. Chronic and progressive side effects may be viewed by patients as a constant reminder of their cancer or be misinterpreted as an indicator of cancer recurrence, which leads to higher FCR scores. Besides, patients may hold doubts about the efficacy of RT and feel afraid that they would not receive the same intensive attention and care from health professionals when at home. For those patients who experience a downward (i.e. negative) fear trajectory, one possible reason is that RT is usually the final treatment they received for their disease (after a long duration of treatment - surgery, chemotherapy and RT) and patients may start to feel confident about their attempt to return to normal life.

Patients in the group who received a boost reported more fears than patients in the routine and DCIS group. One explanation is that patients with a boost may conclude that they have a more serious form of breast cancer which requires more intensive treatment. As mentioned previously this is not the case and there may be an argument in favour of sharing the issue of margins with patients in the decision to perform the additional boost fractionation. DCIS patients report lower FCR levels compared to the other two groups because their clinicians typically explain that this condition is not as serious compared to other forms of breast cancer.

The LGCM analysis showed clearly that the initial level (intercept) was the strongest predictor of follow up FCR into the first 2 months of ‘survivorship’. This finding would tend to support the view that FCR is quite stable and already present at the start of RT. Of interest was the additional explanatory value of the trajectory slope with follow up FCR, independent from baseline. Hence patients who reported increasing levels of FCR during the course of their RT would be more likely to have a higher level of follow up FCR in comparison to those with decreasing FCR, taking into account the initial level of FCR. This finding, if replicated, would be important new knowledge as it would demonstrate that for an important group of patients whose concerns about the cancer returning are already being promoted in some way. It raises a crucial question of whether there might be some intervention with these patients to interrupt such a process. Theoretically, according to Leventhal’s self-regulation model [41], we can speculate that patients may be identifying a particular side effect or symptom that they dwell upon. These illness representations potentially ‘fuel the fire’ of anxieties about cancer returning at an early stage from diagnosis while being treated. Intervention while staff are in close contact may be recommended prior to patient discharge from the service. Some initial support for this interpretation has been identified [42].

### Study limitations

The study was run in a single cancer centre in Scotland limiting generalisation. Of those patients that were initially approached approximately just over half participated. Just under 20% of patients were lost to follow up. They were not significantly different from those that remained on demographic, treatment or initial FCR level variables. One exception out of the 13 tests conducted to examine this drop out issue was that, at the 5% level, patients who were lost to follow up tended not to report additional health problems. Some caution would need to be exercised when interpreting these findings. The DCIS patients were excluded in the LGCM approach as they were a small group of 5 patients and completed additional ratings beyond 4 weeks due to their elongated radiotherapy schedule. The sample size for conducting latent variable estimation limited the control variables other than treatment group assignment. The missing data over the course of the treatment from participants in the study were managed by the statistical approach provided, and an efficient estimation procedure we believe enabled reasonable conclusions to be drawn. A further issue worthy of mention is ‘reactivity’, that is, does inviting participants to rate their FCR weekly sensitize them? We gave no desired expectation for patients, that is encouraging a particular level or FCR end state. One of the experts in the field of diary methods has concluded that multiple assessments, when there is no explicit goal presented to the respondent, ‘does not seem to have a meaningful influence on their thoughts, feelings’ page 8 [43]. A detailed discussion about this issue can be found [44] and tests to investigate in detail in further studies are warranted using ‘intentional missing-data designs’ [45]. In addition future work should concentrate on running more observations of FCR across other centres to confirm that knowing a patient’s FCR trajectory provides additional information to the baseline FCR level to predict eventual FCR level at around 2 months after treatment. The LGCM approach used does not allow for heterogeneity or clustering of individual trajectories. Investigators are encouraged to adopt a growth mixture model (GMM) coupled with LGCM that would enable a more realistic attempt to reflect patterns of trajectories in different ‘classes’ or types of patients [36].

### Clinical implications

Patients whose FCR changes during the course of RT may be identified using simple rating scales or well-chosen open questions at review appointments. Attempts at listening to concerns such as these may be crucial turning points to divert the process of FCR development. The study of these sequential and dynamic processes over time enables the clinical team to consider approaches to intervene and prevent patients reflecting unduly or ruminating on symptoms or illness representations that may be inaccurate.

## Conclusions

FCR at approximately 2 months from final treatment of radiotherapy can be predicted reliably from their baseline FCR rating, i.e. start of RT, and in addition from their FCR trajectory during RT.

## Abbreviations

BT: Brachytherapy
CFI: Comparative fit index
CT: Computed tomography
DCIS: Distal carcinoma in situ
ECC: Edinburgh Cancer Centre
FCR Follow-up: Fears of cancer recurrence total score at Follow-up
FCR: Fears of cancer recurrence
FCR7 Week 1: Fears of cancer recurrence total score at 1st week baseline
FCR7 Week 1: Fears of cancer recurrence total score at 2nd week
FCR7 Week 1: Fears of cancer recurrence total score at 3rd week
FCR7 Week 1: Fears of cancer recurrence total score at 4th week
FCR7: Fears of cancer recurrence scale-7 item version
FIMR: Full information maximum likelihood
GMM: Growth mixture model
LGCM: Latent growth curve model
NHS: National Health Service
NRES: National Research Ethics Service
RT: Radiotherapy
SD: Standard deviation
SRMR: Standardized root-mean-square residual
Tx: Treatment type
